# Bile acids in the fountain of youth

**DOI:** 10.18632/aging.100169

**Published:** 2010-07-16

**Authors:** Gerardo Ferbeyre

**Affiliations:** Département de Biochimie; Université de Montréal; C.P. 6128, Succ. Centre-Ville, Montréal, Québec, H3C 3J7; Canada

Anyone lucky enough to drink water from the fountain of youth could retard its aging process, as the legend tells. Most likely, water was not the only element in the legendary fountain.  What else could be found in there? We may never know! But scientists could come across their own anti-aging ingredients using model organisms and collections of chemical compounds, as the Titorenko's lab reports in this issue of Aging.

Research in simple model organisms clearly indicates that aging can be slowed down by genetic manipulation or dietary intervention.  Astoundingly, a limited set of nutrient- and energy-sensing signaling pathways has emerged as a central regulator of longevity across the evolutionary tree from yeast to mammals [1]. A compilation of these evolutionarily conserved pathways includes the insulin/insulin-like growth factor 1 (IGF-1), AMP-activated protein kinase/target of rapamycin (AMPK/TOR) and cAMP/protein kinase A (cAMP/PKA) pathways [1,2]. None of these pathways are linear as they share several protein kinases and adaptor proteins, thereby being converged into a signaling network regulating longevity [2,3].

The dietary regimens known as caloric restriction (CR) extends longevity across species by causing a specific remodeling of the aging signaling network [1,4]. Certain small molecules can provide the benefits associated with CR and are known as "CR mimetics" [5]. Most of these compounds are unable to extend life span if the supply of calories or nutrients is limited suggesting that they are acting in the same signaling pathways [6,7,8]. In contrast, Li^+^ can extend life span in worms and rapamycin in fruits flies even under CR conditions [9,10]. Titorenko and colleagues (Goldberg et al.) suggested the existence of "constitutive" or "housekeeping" longevity pathways that can operate irrespective of the organismal nutrient and energy status [11]. In quest for the envisioned housekeeping longevity pathways, they carried out a chemical genetic screen for small molecules that increase the chronological life span (CLS) of yeast under CR conditions.

It turns out that one of the most potent anti-aging compounds identified by Goldberg et al. in their chemical genetic screen was lithocholic acid (LCA), a bile acid [11]. Interestingly, none of the currently known life-extending molecules is structurally related to LCA and none of them was able to extend the CLS of a short-lived yeast mutant which Goldberg and colleagues used in their screen for compounds specifically targeting housekeeping longevity pathway(s). The finding that LCA modulates yeast life span is however, surprising, given the fact that yeast does not synthesize LCA or any related compound [12,13].

**Figure 1.**
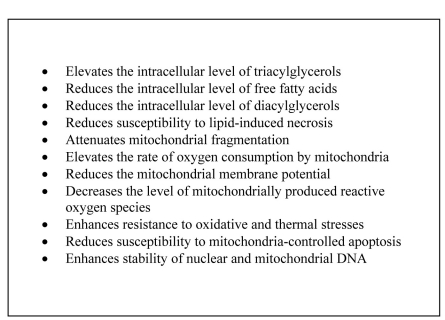
The pleiotropic effect of LCA on various longevity-related processes in yeast.

Although the mechanistic details are yet unknown, Goldberg and colleagues found that LCA influences various longevity-related processes.  First, LCA acts in a calorie availability-independent fashion and includes several anti-aging mechanisms (Figure 1). Second, LCA unmasks a previously unknown anti-aging potential of PKA, a key player in the cAMP/PKA pathway. Interestingly, bile acids modulate potential antiaging pathways in mammals as well [12,13] and accumulate in the serum of long living little mice. They could modulate the activity of nuclear receptors controlling the expression of genes of xenobiotic metabolism [16]. Goldberg et al. propose that LCA is a xenobiotic regulator of aging in yeast acting mainly as a mild toxic compound that triggers endogenous cellular longevity pathways. The idea is consistent with recent studies in mice indicating that promoting chemical hormesis with molecules having detergent-like properties such as bile acids may extend life span [14,15].

By providing important new insights into mechanisms of longevity regulation by a novel anti-aging compound, the study of Goldberg et al. raises important questions for future research. Perhaps the most critical is to elucidate the direct cellular targets of LCA, responsible for the variety of antiaging pathways triggered by this compound. A genetic screening for mutations capable of extending life span under CR conditions will probably bring additional insights into housekeeping longevity assurance pathways. Mutations in the glucose-sensing pathway of *S. pombe* increased life span independently of the metabolic effects of glucose and actually cooperated with CR [17]. Hence, glucose signaling may be modulated by housekeeping longevity assurance pathways.

Although we should not start taking bile acid supplements yet to live longer, further research on the effect of bile acids on the diseases associated to old age may offer a hope to live a healthy aging.
